# Cementitious Grouts for Semi-Flexible Pavement Surfaces—A Review

**DOI:** 10.3390/ma15155466

**Published:** 2022-08-08

**Authors:** Muhammad Imran Khan, Muslich Hartadi Sutanto, Nur Izzi Md. Yusoff, Salah E. Zoorob, Waqas Rafiq, Mujahid Ali, Roman Fediuk, Nikolai Ivanovich Vatin

**Affiliations:** 1 Department of Transportation & Geotechnical Engineering, National University of Sciences and Technology (NUST), Islamabad 44000, Pakistan; 2 Department of Civil & Environmental Engineering, Universiti Teknologi PETRONAS, Seri Iskandar 32610, Malaysia; 3 Department of Civil Engineering, Universiti Kebangsaan Malaysia, Bangi 43600, Malaysia; 4 Construction and Building Materials Program, Kuwait Institute for Scientific Research, Safat 13109, Kuwait; 5 Department of Civil Engineering, Faculty of Engineering, Universiti Malaya, Kuala Lumpur 50603, Malaysia; 6 Polytechnic Institute, Far Eastern Federal University, 690922 Vladivostok, Russia; 7 Peter the Great St. Petersburg Polytechnic University, 195251 St. Petersburg, Russia

**Keywords:** cementitious grout, grouted macadam, porous asphalt mixture, semi-flexible pavement, grouting ability

## Abstract

The hybrid type of pavement called semi-flexible or grouted macadam has gained popularity over the last few decades in various countries, as it provides significant advantages over both rigid and conventional flexible pavements. The semi-flexible pavement surface consists of an open-graded asphalt mixture with high percentage voids into which flowable cementitious slurry is allowed to penetrate due to gravitational effect. Several researchers have conducted laboratory, as well as field, experiments on evaluating the performance of semi-flexible layers using different compositions of cementitious grouts. The composition of grouts (i.e., water/cement ratio, superplasticizer, polymers, admixtures, and other supplementary materials) has a significant effect on the performance of grouts and semi-flexible mixtures. A comprehensive review of cementitious grouts and their effect on the performance of semi-flexible layers are presented and summarized in this review study. The effect of byproducts and other admixtures/additives on the mechanical properties of grouts are also discussed. Finally, recommendations on the composition of cementitious grouts have been suggested.

## 1. Introduction

Pavement construction aims to sustain traffic loads and environmental stresses throughout its design life. Good riding quality, durability, fatigue, and rutting characteristics are some of the key criteria that are considered during a comprehensive pavement design process. Traditionally, there have been two main choices of road construction: flexible and rigid pavements. Flexible pavements are constructed from bituminous materials, whereas rigid pavements are constructed from concrete. Conventional flexible pavements are more susceptible to rutting and fatigue distresses when subjected to heavy traffic loading and adverse weather conditions [1]. The service life of the flexible pavement is relatively short and frequent maintenance is required to preserve the pavement condition. Furthermore, routine maintenance of flexible pavement increases its overall cost. The distresses in the flexible pavement are mainly due to choice of material selection, poor mix design, and compromise on quality during construction, as well as overloading issues. On the other hand, rigid pavements have high construction costs and are usually constructed for specialized road types. Rigid pavement construction and opening to traffic usually take a longer time due to the slow concrete setting. Furthermore, rigid pavement is also avoided due to rough riding quality and provision of joints that are provided to relieve the thermal stresses.

Therefore, a new hybrid type of pavement, which is the main subject of this review study, has gained popularity in the last few decades. It is known as semi-flexible pavement, where the surface layer incorporates a semi-flexible material. It can combine the best characteristics that flexible and rigid pavements contain separately. The semi-flexible pavement surfacing, also known as grouted macadam, is believed to have a long design life, significant resistance to rutting, high durability, and joints-free pavement with an impermeable surface [2,3]. It can provide resistance against fuel if constructed in an area exposed to fuel spillage (such as oil refineries, fuel stations, and airport aprons, etc.).

The semi-flexible pavement surfacings are comprised of an open-graded asphalt skeleton (with 25–35% voids), over which a highly flowable cementitious grout is spread and allowed to infiltrate [4,5]. Sufficient flowability of the cement grouts to fill the voids and the interconnectivity of voids are considered the critical properties of semi-flexible pavement surfacings. These surfacings can be used for heavy-loaded pavement sections, as they provide excellent resistance to rutting. They can also be used in airports, industrial areas, fuel stations, and refineries as a load-bearing and fuel-resistant surfacing. The following sections present a brief overview of research performed in the modification and improvement of binder and mixtures for flexible pavements, and then a detailed overview of cementitious grouts for semi-flexible pavement and their performance is presented. 

## 2. Review on Modification of Asphalt Binder and Mixes for Conventional Flexible Pavements

Before moving on to semi-flexible mixtures, it is quite important to discuss the failures caused in conventional flexible pavements and the methods of improving their performance. The distresses in the pavements are the result of continuous and cumulative damage in different pavement layers caused by repeated traffic load and adverse weather conditions. Some very common distresses in flexible pavements are cracks (fatigue, longitudinal, transverse, block, reflective, and edge cracks), deformation (rutting, corrugation, showing, and depression), deterioration (potholes, raveling, stripping, polished aggregate, and pumping), mat problem (segregation and bleeding) and seal coats (rock loss, segregation, bleeding, fat spot, and delamination) [6]. Studies by various researchers reveal that the main reason behind such types of failures are due to the following: heavy traffic load, high temperatures, water infiltration, hot and humid weather, freezing temperatures, poor quality of materials (aggregate, asphalt binder, and soil), as well as poor mix design and construction practices [7,8,9]. 

Performance improvement of roads in terms of new materials is a common practice in the construction of local, as well as major, highways. Various layers of the road may need improvement, depending on the type of road, as well as the availability and characteristics of local materials. Among these layers, the sub-grade and the top hot mix asphalt (HMA) layers are the most important and critical layers. If the sub-grade deforms, the rest of the layers also exhibit deformation, and this leads to the failure of the pavement. The HMA layer, which is directly subjected to traffic and environmental stresses, needs careful treatment to improve its performance. Several techniques have been recommended by researchers to stabilize different layers of pavement around the globe. The properties of weak sub-grade soil have been significantly improved using stabilizing materials, such as: oil fuel fly ash (FFA), cement, foamed asphalt (FA) and foamed sulfur asphalt (FSA), liquid asphalt, hydrated lime, synthetic fibers, a geotextile layer, and polypropylene fibers [10,11,12,13,14,15,16]. Many professionals have conducted considerable research on modifications of conventional asphalt using different polymers and waste materials. A study was conducted using waste oil fly ash (OFA) as an asphalt modification. To improve the bonding with asphalt, OFA was functionalized using different chemicals, such as the carboxylic group (-COOH), the amine group (-NH_2_/NH), and the C18 group (C_18_H_38_O). The treated OFA-modified binder showed improved performance in terms of reducing temperature susceptibility, increasing performance grade, increasing rutting resistance, and better resistance to low-temperature cracks, compared to the untreated OFA-modified binder [17,18,19]. 

Much literature is available related to the improvement of properties of asphalt binder and mixtures. However, very limited research is conducted on grouts and semi-flexible pavement layers. Many researchers have used crumb rubber as a bitumen modifier, and the performance in terms of fatigue life, rutting resistance, resistance to oxidation, and ageing and moisture susceptibility were evaluated [20,21,22]. Research was conducted on utilizing waste crumb rubber in the performance evaluation of sulfur asphalt. The rheological properties were investigated, and it was concluded that the addition of crumb rubber to sulfur asphalt increased the elastic properties, zero shear viscosity, and rutting parameter (G*/sinδ) [23]. Crumb rubber (CR) can also be used to modify the locally available base asphalt because of its rheological properties. It was concluded that there is a considerable increase in complex modulus (G*) and a reduction in the phase angle (δ) with an increase in CR percentage at a high temperature, which is an indication of improving the elastic properties of CR-modified binders [1,24,25]. However, it was not effective in improving the fatigue of asphalt mixes when dense aggregate gradation was used [26].

Plastics are used in a wide range of products and plays an essential role in our daily life. It is due to a high strength-to-weight ratio, low cost, and easy availability of plastics everywhere. Most of these plastics are not biodegradable by nature, which contributes to environmental concerns. Despite awareness and efforts, plastic recycling after usage is pretty low. It has been reported that about 8.8% of waste plastic is recycled, while the rest of waste plastic is simply dumped into landfill and, hence, is a serious threat to human health and the environment [27,28]. Hassani et al. [29] researched the use of Poly-Ethylene Terephthalate (PET) as a modifier in an asphalt concrete mixture as an aggregate replacement. They concluded that there is a 2.8% reduction in bulk compacted mix density with an aggregate replacement of 20% by volume with Polyethylene Terephthalate (PET) granules. It was also concluded that Marshall Stability and Marshall Quotient for the control mixtures and PET-modified mixtures (used as a partial aggregate replacement) were almost the same. Recycling of PET with asphalt meets most of the specification’s requirements that make it appropriate for use in road construction, and the recycling of PET also reduces environmental pollution [30]. It was concluded that natural rubber and crumb rubber are sustainable modifiers and they have been used for a wide range of asphalt modification for a cost-effective, rutting, and pavement fatigue resistance. Results show that with an increasing percentage of CR in the mixture of stone mastic asphalt (SMA), the compaction effort is increased, and drainage of the binder is decreased to fulfil the volumetric properties of SMA [31,32]. Therefore, researchers are looking for more durable and sustainable pavement surfaces that can provide satisfactory performance and serviceability throughout their design life.

## 3. History of Semi-Flexible Pavement

In the early stages of developing semi-flexible pavement surfaces, researchers and agencies gave different brand names for the same construction and working principles. The first semi-flexible pavement was constructed in the 1950s in France as a fuel and abrasion-resistant surfacing, and was given the name Salviacim [33]. In the 1970s and 1980s, semi-flexible pavement construction spread throughout Europe, the South Pacific, the Far East, several countries in Africa, and North America [34]. Table 1 shows the early development of semi-flexible pavement in different countries with different brand names.

## 4. Semi-Flexible Pavement/Grouted Macadam

**Figure 1:**
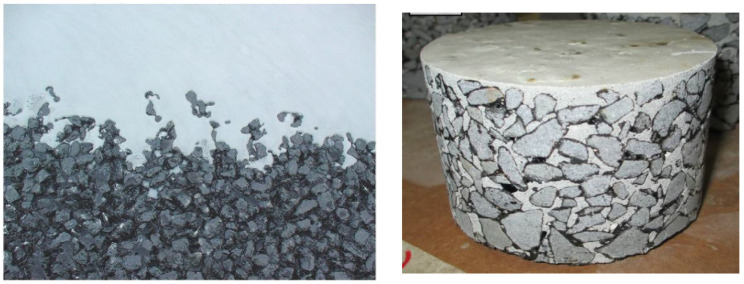
Grouted Macadam (semi-flexible pavement surface/mixtures) [4,45,48].

Semi-flexible pavement consists of an open-graded asphalt structure with 20 to 35% voids into which flowable cementitious slurry is allowed to penetrate due to pouring and gravitational effects, as shown in Figure 1. Conventionally, Portland cement grout has been used as a cementitious slurry by pouring into open-graded asphalt mixtures, constructing a semi-flexible layer. Numerous researchers have conducted extensive laboratory, as well as field, experiments on evaluating the performance of semi-flexible pavement, which showed significant improvement in compressive strength, stability, and durability [41,42]. Moreover, a semi-flexible surfacing combines the flexibility of asphalt with the rigidity of the cement grout. The rigid cement grout provides high compressive strength, high durability, and resistance to wear. It has been practiced on the bus lanes in urban areas [43]. Based on an extensive review by Du et al. [44], it has been proposed that a semi-flexible pavement layer could be a better solution against rutting distresses [45].

A significant component in a semi-flexible layer is the cementitious grout, which contributes to rigidity and durability, with high compressive strength. The cementitious grouts need to be highly flowable to penetrate fully into the porous asphalt skeleton. The grouts may consist of cement, water, superplasticizer, admixtures, and/or other supplementary materials. Superplasticizer and admixtures/other supplementary materials are required to achieve the required flowability without compromise on the strength properties of grouts. Various combinations of cementitious grouts, such as mild glass, Panasqueira Waste Mud, and geopolymeric grouts, were investigated to evaluate the performance of a semi-flexible pavement surface [46]. Grouts play a significant role in the performance of flexible pavements. The modified grouts showed improved results in Marshall stability, stiffness, compressive strength, and resistance against rutting [46]. Furthermore, this type of pavement surface has been developed to provide rut resistance and joints-free pavement. Initially, it was developed for heavy-loaded sections with slow-moving or static loads, particularly at intersections, for rut resistance without cracks and joints [47]. 

Depending on the mix design of a porous asphalt structure and grout filling, the semi-flexible pavement surface shows variability in its mechanical properties. A study was conducted to compare the fatigue performance of grouted macadam with that of a conventional blend, using the same percentage of asphalt and type of aggregate. It was revealed that the average stiffness modulus of the grouted mixture was approximately three-times higher than that of a conventional asphalt mixture [48]. A study was conducted to optimize the above-stated characteristics for cement paste (containing cement, coal ash, mineral powder, and water) and cement mortar (containing cement, coal ash, mineral powder, fine standard sand, and water) as a cementitious grout for semi-flexible pavement surfaces. Cement paste (optimum value with 0.56–0.58 water–cement ratio, 10% fly ash, and 10% mineral powder) exhibit better overall performance compared to cement mortar (optimum design with 0.61–0.63 water–cement ratio, 10% fly ash and 15% fine sand), though both grouts meet the recommended technical requirements within the recommended mixture ratio range [49]. In general, the cementitious grout for a semi-flexible pavement surface should have sufficient flexure and compressive strength, as well as better fluidity, ductility, and resistance to drying shrinkage.

Resin-Modified Pavement (RMP) was used as a grouted macadam and was suggested to be used in the rehabilitation of existing pavement, as well as in new pavement construction. RMP consists of a porous asphalt mixture with 25–35% voids and cementitious grouts (containing: cement, silica sand, fly ash, water, and resin additives). It was considered an alternative to the rigid pavement, as it has a low construction costs and can be used in any environmental conditions [36]. The layers beneath the grouted macadam layer are protected due to its impervious nature (as voids are filled with cement grout), and the stress level in the base course is significantly reduced. The speed of construction and time required to open for traffic is significantly improved compared to rigid pavements [50]. Another important property of semi-flexible pavement surfaces is the early gain in strength and the smaller amount of time required to achieve the final properties of the layer compared to rigid pavement. This type of pavement can be opened to traffic after two to three days, while a rigid pavement may need 21 to 28 days [40]. 

## 5. Factors Influencing the Performance of Grouts and Semi-Flexible Mixtures

The semi-flexible mixture/layer consists of a porous asphalt skeleton and cementitious grouts, and hence, the performance of the semi-flexible layer depends on both components. Some of the factors influencing the performance of semi-flexible layers are identified and discussed as follows.

### 5.1. Porous Asphalt Mixture Gradation

**Figure 2:**
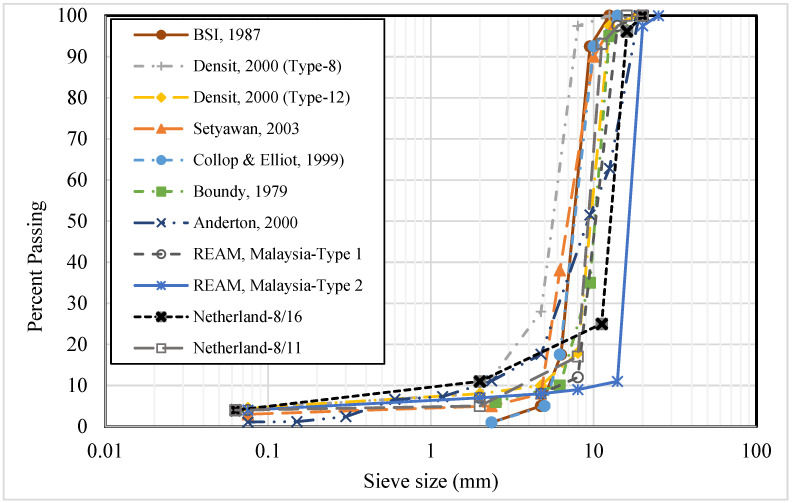
Porous aggregate gradation used in past studies.

The selection of aggregate gradation plays a significant role in the performance of the final mix’s properties. Usually, a single-sized aggregate in open-graded asphalt gradation is used for the preparation of porous asphalt mixtures. Generally, 90–95% coarse aggregate, 4–5% sand, and 2–4% filler are used in the design of open-graded asphalt mixture for semi-flexible pavement applications [51,52]. The selected gradation should provide air voids of 25 to 35% in the open-graded asphalt mixture, to allow cementitious grouts to penetrate the layer fully. As the open-graded asphalt gradation contains high percentages of course aggregate, but very low percentages of fines and fillers, a low quantity of bitumen (2 to 4% by weight aggregates) is therefore used, due to less surface area [51]. Various researchers have adopted different gradation systems in the past for the mix design of the porous asphalt skeleton, as shown in Figure 2. It can be seen that the main difference is the nominal size, between 8 to 14 mm, and most of the gradation is single-sized, with very low percentages of fine aggregates, which makes the porous asphalt skeleton. However, the common goal is to achieve the target 25–35% air voids in the mixture, in order to ease the quick infiltration of grouts due to void interconnectivity and high porosity [40,53,54,55]. Nevertheless, in Malaysian specifications, the nominal size is 20 mm for type-1 and 14 mm for type-2 gradation systems used in the porous asphalt skeleton for semi-flexible pavement surfaces [56]. In the Netherlands, the nominal size for the porous asphalt skeleton is 11.2 mm for type 8/11 and 16 mm for type 8/16 gradation systems [57]. 

**Figure 3:**
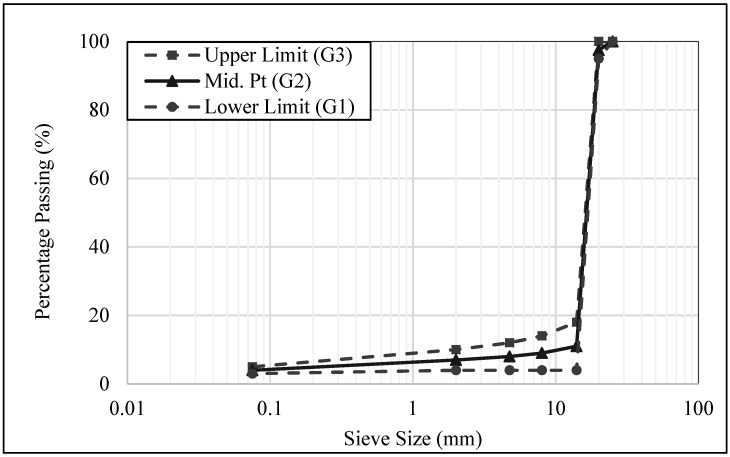
Aggregate gradation adopted from the Road Engineering Association Malaysia (REAM) Type-2 [56].

A study was conducted on using REAM type-2 porous gradations with upper, lower, and midpoints of the gradation limits, as shown in Figure 3. The effect of these gradations on the volumetric and performance properties of semi-flexible mixtures was investigated. It is clear from Table 2 that different aggregate gradations of the porous asphalt skeleton have a significant effect on the resilient modulus and compressive strength properties of semi-flexible specimens. The higher the resilient modulus, the greater the material resistance against permanent deformation. Similarly, the three aggregate gradations have a different compressive strength of semi-flexible specimens using the same type of grout [58]. Furthermore, aggregate gradation (G3) with less porosity causes a delay in grout infiltration, and hence, a smaller amount of grout was utilized, due to which G3-based, semi-flexible specimens present the lowest compressive strength and least resistance to abrasion compared to G1 and G2 aggregate gradation [59]. A single-size aggregate in the range of 10–14 mm with very small percentages of fines can produce a porosity of 25 to 35% in mixtures, which allows cement slurry to penetrate easily due to interconnected voids. In other words, the aggregate gradation of a porous asphalt skeleton has a significant effect on the performance of semi-flexible pavements. 

Similarly, a study was conducted by Saboo et al. (2019) to rank different types of porous asphalt skeleton for semi-flexible pavement, by developing a hierarchical ranking strategy [51]. The detail of seven different selected porous asphalt mixes and the evaluation criteria are presented in Table 3 and Table 4, respectively. A bitumen content of 2 to 4.5% was used for all gradations (M1 to M7).

Initially, 42 mixes were prepared and evaluated for draindown, air voids, VCA, permeability, and cantabro loss. Cantabro loss is reported by measuring the percentage loss of the mass of the specimen after being subjected to 300 revolutions (at 30 rpm) in a Los Angeles abrasion machine [51]. Only eight mixes fulfilled the criteria and were further evaluated for ITS; however, finally, based on the hierarchical ranking methodology, only three mixes (Densiphalt type 12 with 4.0% bitumen, Densiphalt type 12 with 4.5% bitumen, and BSI with 4.0% bitumen) were selected as the most desirable porous asphalt mixtures that can be used for semi-flexible pavement surfaces [51]. 

### 5.2. Porosity/Voids of Asphalt Mixture

The voids ratio, or porosity, of open-graded asphalt mixture has a significant effect on the properties of the final semi-flexible mixture. Hence, with too low porosity, the voids may not be filled completely with grout, due to poor connectivity in the voids, while high porosity requires a sufficient amount of grouts, and the pavement acts as a rigid structure [60]. The study revealed that an increase in void content (from 20 to 26%) in the porous asphalt skeleton results in an improvement in the compressive, flexure, and splitting strength and resilient modulus of semi-flexible specimens, because more cementitious grout is required to fill the voids, which impart rigidity to final mixtures. However, a slight reduction in compressive strength and resilient modulus was witnessed by increasing voids beyond 26%. Moreover, the increase in void content causes a reduction in Marshall stability and an increase in the flow value of porous asphalt specimens [61]. This reduction in the stability and increase in flow value is due to a reduction in compacted density with increasing porosity. 

### 5.3. Degree of Grout Saturation

The grouting ability of the semi-flexible mixture is measured by determining the degree of grout saturation. It indicates how many air voids (pores) of open-graded asphalt mixture are being filled with the grouts [62]. It is one of the important parameters that may influence a semi-flexible mixture’s performance when subjected to heavy traffic loading and poor weather conditions [63,64]. According to the Chinese standards, the degree of grout saturation should be ≥92% [64]. The degree of grout saturation is not only a function of air voids in the porous asphalt mixture, but also dependent on the voids’ morphological characteristics, size, and structure, which directly affect the interconnectivity of voids in porous asphalt mixtures [65]. 

### 5.4. Type of Asphalt Binder

**Figure 4:**
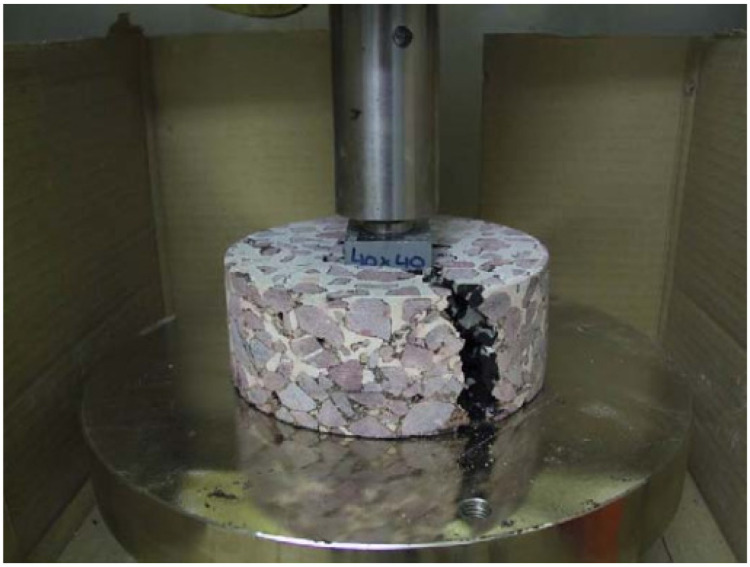
Test setup of indentation strength [57].

The type of asphalt binder in a porous asphalt mixture has a substantial effect on the properties of the semi-flexible layer. Using stiffer and harder asphalt, the stiffness and resistance against the indentation compressive strength of grouted macadam increases, even at high temperatures [57,66]. The test setup of indentation compressive strength is given in Figure 4.

However, stiffer binder causes cracks in the pavement after repetition of traffic loads [60]. The effect of modified and conventional binders on the performance properties of semi-flexible specimens was analyzed. The modified binder has a notable effect on the indentation strength of semi-flexible specimens at 20 °C, and significant improvement was witnessed in the modification of conventional binder with polymers; however, similar indentation strength was noticed at 40 °C [60]. However, the selection of the type of binder is dependent on weather conditions and site location [67]. Therefore, using polymer-modified asphalt with improved higher and lower temperature performance grades (PG) is a better choice to counter the effect of hot and cold temperatures, respectively. 

### 5.5. Water–Cement (w/c) Ratio of Grout

In semi-flexible pavement surfaces, the cementitious grouts are required to be highly fluid to fully penetrate the porous asphalt structure. Hence, the water/cement (w/c) ratio plays a key role in developing the strength and fluidity of cementitious grouts. Different combinations of water/cement ratios (0.45, 0.50, 0.55, and 0.60) were used to optimize the w/c ratio based on compressive strength at 7-days, 28-days, 56-days, and 90-days curing. The target strength of 15–35 MPa on 7-days curing was achieved at a 0.50 w/c ratio [42]. Khan et al. utilized a response surface methodology to optimize w/c ratio and a dose of superplasticizer to formulate cementitious grouts for semi-flexible pavement surface applications [68]. In another study, the water–cement ratio of 0.68 was achieved as the optimum ratio for Latex Resin-modified grouts based on the marsh flow cone viscosity test [69]. This amount of water produced high flowable grouts and fully penetrated the porous asphalt with a considerable durable and tough surface to resist heavy traffic load. A similar and close range of water–cement ratio (0.51 to 0.57) has been recommended based on fluidity, drying shrinkage, ductility, compressive and flexure strength, while the cement grout was modified with a polycarboxylate superplasticizer, expansion admixture, and triterpenoid saponin air-entraining agent additives (details are shown in Table 4 and Table 5) [70]. 

The most important factor that can affect the mechanical properties of grouts and semi-flexible specimens is the fluidity (flow time) of cementitious grout to fill the voids, and hence the porosity and water–cement ratio. It was observed in a study that the porosity of the porous asphalt skeleton and flow time of grouts should be 20% and 12 s, respectively, to achieve the full penetration of grouts. However, to improve the performance properties of grouts and semi-flexible specimens, a chemical admixture (Polyacrylate Polymer) and accelerating and retarding agents were also added to grouts in different proportions [71]. So far, limited literature is available on optimizing the composition of grouts based on the physical and performance analysis of grouts and semi-flexible specimens. Based on a previous research study, a water–cement ratio in the range of 0.45 to 0.63 can be used to achieve the required fluidity and strength properties of grouts [70,72,73,74]. 

The effect of using waste tire rubber as a cement powder replacement in grouts was studied, and it was concluded that, with a w/c ratio of 0.60, ordinary Portland cement (OPC) should not be replaced by more than 20% of waste tire rubber. A small addition of tire rubber caused a reduction in compressive and flexure strength; however, the performance against low temperatures can be improved with the addition of tire rubber [75]. In short, an increase in the w/c ratio has an inverse relation with strength properties (compressive and flexural strength); the higher the w/c ratio, the less strong, and vice versa. The w/c ratio can be controlled by adding superplasticizer to improve the fluidity of grout, without compromising the strength properties [76]. The workability of grouts can be significantly improved by adding a superplasticizer [77]. Most commonly, polycarboxylate ether- superplasticizers were used in various studies [70,78,79,80]. 

### 5.6. Admixtures and Other Supplementary Cementitious Materials

The composition of grouts (i.e., w/c ratio, superplasticizer, polymers, and other modifying agents) has a significant effect on the performance of grouted macadam. Minimal research literature is available on different compositions and the performance of cementitious grouts for semi-flexible pavement surfaces. Controlling the viscosity of cementitious grout plays a vital role in achieving full penetration of voids in the porous asphalt skeleton and the target strength of the mixture. If the fluidity of grout is low, some voids may not be filled at the bottom of the specimen. On the other hand, if the w/c ratio of grout is high, a reduction in the overall strength of the mixture occurs. Superplasticizer can be used at a relatively low w/c ratio to achieve high fluidity [81]. Similarly, the type and concentration of superplasticizer influence the flow and strength properties of grouts. Two types of Polycarboxylic ether polymer and one type of sulphonated naphthalene formaldehyde with different dosage were used as superplasticizers to investigate flow and compressive strength. The results reveal that Polycarboxylic ether superplasticizer is superior in achieving the target flow and strength properties [82]. Malaysian specifications recommend that the target flow of 11 to 16 s (using Malaysian flow cone) and target compressive strength of 50 MPa at 1-day and 105 MPa at 28-days of cementitious grouts (grouts containing silica fume, superplasticizer, admixtures) should be used in constructing semi-flexible pavement surfaces for heavy-loaded sections [56,83]. 

A study was conducted by using Carboxyl latex (0 to 12%) as a partial replacement of cement in cementitious grout for a semi-flexible layer. The design parameters, water/cement ratio (0.63), mineral fillers (10%), and sand (20%) were selected based on a literature study. The optimum asphalt content (OAC) was obtained by a Marshall stability test, cantabro, and drainage test. It was concluded that cement grout containing 8% carboxyl latex showed improvement in flexure strength and better volume stability. In addition, the performance against rutting at high temperatures, low-temperature cracks, moisture damage, and fatigue were significantly improved in grout modified with carboxyl latex [72]. Different proportions of water–cement ratio, superplasticizer, and sand quantity in making grout for semi-flexible mixtures were studied. It was concluded that rupture and compressive strength were higher, but the fluidity was affected in reducing the w/c ratio from 0.3 to 0.21. However, a 0.22 w/c ratio, 0.3% fine sand, and 0.01% superplasticizer give the best combination in terms of fluidity, and compressive and rupture strength [84]. The fluidity of grouts increased with an increase in the water–cement ratio, while a reduction in compression strength and flexure strength was observed. The water–cement ratio in the range of 0.55 to 0.57 has been proposed for ordinary cement paste in a semi-flexible layer [84,85]; in addition, the acceptable ranges of using polycarboxylate superplasticizer content, expansion admixture, and air-entrained agents are 0.5–1.0%, 10%, and 0.008%, respectively, have been proposed for high-performance cement paste as a grouting material for semi-flexible pavement surfaces.

In another study, three types of grouts were used, namely, Densit grout (commercially available in the UK), Densit-modified grout, and weak grout (Densit + 25% silt). However, no significant change in the stiffness modulus and phase angle of grouted macadam was observed. However, a slight improvement in the case of Densit grout was noticed. Furthermore, grout strength and grout shrinkage are considered two characteristics used to analyze the fatigue performance of grouted macadam. The Densit grout showed the best fatigue life of macadam, while the modified grout exhibited slightly lower fatigue performance [40]. Similarly, additives and/or admixtures can be used to partially replace cement in cementitious grouts to reduce the overall cost of construction and achieve the target strength. So far, semi-flexible pavement surfaces are being designed for heavy-loaded sections, and hence, high-strength admixtures or other high-strength supplementary cement materials are necessary to be added in cementitious grouts to achieve high compressive strength (50 MPa at 1-day and 105 MPa at 28-days curing) with acceptable fluidity [56].

### 5.7. Industrial/Municipal Wastes and Other Polymers/Additives

The cementitious grouts contribute to making a semi-flexible layer rigid and durable. However, utilizing OPC in grouts for the construction of semi-flexible pavement is a severe threat to the environment in terms of carbon dioxide emissions. The cement industry accounts for carbon dioxide emissions, making it a critical sector for emission mitigation. The production of cement releases carbon dioxide both directly and indirectly. Furthermore, the high cost of cement requires a huge investment in constructing semi-flexible pavement surfaces using OPC grouts. Hence, researchers are investigating the use of alternative cementing materials or the partial replacement of cement with industrial/municipal wastes or byproducts to control or minimize the use of OPC in the construction industry [86,87]. 

Another study was conducted on utilizing fly ash and mineral powder (ground granulated blast furnace slag) in cementitious grouts for semi-flexible mixtures. Two types of grouts, namely, cement paste (cement, fly ash, mineral powder, and water) and cement mortar (cement, fly ash, mineral powder, standard sand, and water), were prepared. In this study, the optimum contents of fly ash, mineral powder (ground granulated blast furnace slag) and water–cement ratio for the cement paste were 10%, 10%, and 0.56–0.58, respectively, based on evaluating the fluidity, ductility, compressive and flexure strength (7, 28-days) and drying shrinkage properties. Similarly, for cement mortar, the optimum contents of fly ash, mineral powder, standard sand, and w/c ratio were determined as 10%, 0%, 15%, and 0.61–0.63, respectively, using the same methodology as adopted for cement paste. However, at a certain water–cement ratio, the fluidity, compressive and flexure strength of the cement paste were better than the cement mortar. The cement paste grouts with a water–cement ratio, fly ash, and mineral powder contents of 0.58, 10%, and 10%, respectively, were considered optimum compositions for semi-flexible mixtures [49]. 

Various combinations of cementitious grouts, such as mild glass (from recycled bottles), Panasqueira Waste Mud, and geopolymeric grouts, were investigated to evaluate the performance of semi-flexible pavement surfaces. Grouts play a significant role in the performance of flexible pavements. The modified grouts showed improved results of Marshall stability, stiffness, compressive strength, and resistance against rutting [46]. In another study, three types of cementitious grouts were used to evaluate the effect of different types of grouts on the performance of the semi-flexible layer. These grouts were standard Densit^®^ grout (commercially available), modified grout, and weaker grout (obtained by adding 25% silt to Densit^®^). The water–cement ratio of 17.2% by mass of powder was used as a guideline throughout the project. The overall performance of Densit^®^ grouts and hence the Densit^®^ macadam were better than modified and weaker grouts in terms of compressive and fatigue strength, stiffness modulus, resistance to permanent deformation, shrinkage, and thermal cracks [40]. In another study, it was revealed that the addition of silica fume significantly improves the compressive strength of grouts, while reducing the fluidity [81]. Silica fume and fly ash substitution in cement grouts also contributes in the improvement of performance of cementitious grouts [88]. Furthermore, the target compressive strength of 90–120 MPa at 28 days was achieved when cement was replaced with pozzolanic materials such as silica fume and fly ash-based grouts, which also contribute to higher modulus of elasticity and lower porosity of grouted macadam [89]. 

Similarly, cross polymer resin (5 parts water + 2 parts styrene-butadiene + 1part water-reducing agent) was used to produce resin-modified pavement (RMP) mixtures. The slurry grout compositions used in this study include: cement, silica sand (18–18.5%), fly ash (18–18.5%), Latex Resin (2–3%), and water (w/c ratio 0.60–0.70). The grout compositions (such as 36.2% cement, 18.1% sand, 18.1% fly ash, 24.8% water, and 2.8% Latex Resin) were optimized based on a marsh flow cone viscosity test and were used for further field evaluation. The results from field trial sections reveal that RMP could be used as a durable and tough surfacing for military-purpose pavements, as well as for heavy vehicular loads. However, RMP does not provide significant resistance against fuel and oil damage [69]. Similarly, user guidelines were developed in 1996 by Anderton to design RMP mixtures and pavement surfaces. According to the guidelines, the composition of grouts, mix design, and performance properties, as shown in Table 5, are used for RMP mixtures [90]. Ease in construction, no joints on the surface, cost competitiveness, resistance to rutting, suited for any environmental conditions, abrasion, and wear resistance are some of the benefits of RMP. 

**Table 5:** Grout composition and porous asphalt job mix formula for RMP [90].

Grouts Composition		Porous Asphalt	
Material	Weight (%)	Voids	25 to 35%
Cement (Type-1)	34 to 40	Bitumen Content	3.5 to 4.5% by total weight
Sand (silica Sand)	16 to 20	Penetration grade	40 to 100
Fly Ash (as Filler)	16 to 20		
Latex Resin	22 to 26		
Water	2.5 to 3.5 [w/c = 0.65–0.70, Flow time: 8 to 10 s]		

Various combinations of cementitious grouts were investigated from three different types of additives (Th-98 Polycarboxylate Superplasticizer, UEA Expansion Admixture, and ZY-99 Triterpenoid Saponins Air-entraining agent) and recommendations are made based on fluidity, ductility, drying shrinkage, and compressive and flexure strength tests, as described in Table 6 [70]. 

Based on the above-mentioned testing criteria, high-performance cement pasts (H1, H2, and H3) were designed for further investigation, and H2 and H3 were recommended as high early-strength cementitious grouts. The grout compositions for H1, H2, and H3 are expressed in Table 7. 

**Figure 5:**
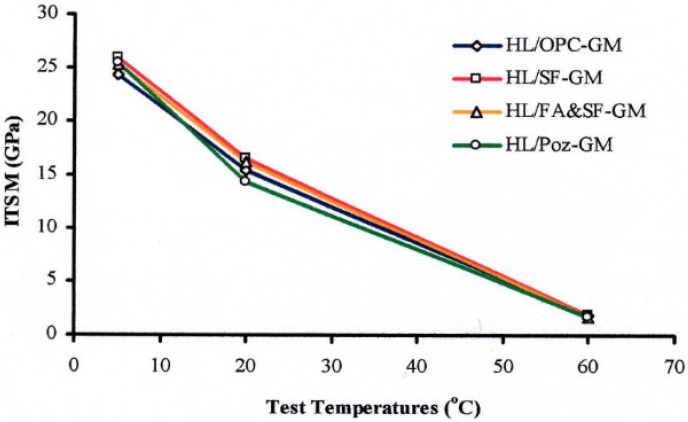
Effect of cementitious grout on the tensile modulus of elasticity [50].

A study revealed that the target performance of semi-flexible specimens could be achieved by controlling the performance and fluidity of grouts. The addition of Polyacrylate polymer, and accelerating and retarding agents significantly improves the fluidity and performance properties of grouts and semi-flexible specimens [71]. Chemical admixtures referred to as superplasticizers or resin modifiers are usually used to make the cementitious grout highly flowable at a low amount of water [36,50,91]. However, using an excess content of superplasticizer can cause bleeding and segregation in cementitious grouts [92]. The early strength of grout can also be improved by using accelerators [50]. Different combinations of OPC, silica fume (SM), and fly ash (FA) were used by Setyawan, 2003 in his study, and no significant difference in stiffness modulus was observed at different test temperatures, as shown in Figure 5 [50].

## 6. Evaluation of Physical and Mechanical Properties of Cementitious Grouts

Various testing techniques have been used to evaluate the physical and mechanical properties of cementitious grouts. The most significant property of grout is to achieve adequate workability or flowability so that grout can easily and fully penetrate the porous asphalt skeleton in achieving the targeted strength. Various factors, including water–cement ratio, type and dosage of superplasticizer, type of cement, and other admixture or supplementary materials, affect the workability of grouts.

The physical and mechanical properties of cement grouts are highly dependent on the composition of the grout. The grouts can be evaluated for flowability, drying shrinkage, compressive strength (at different curing ages), and flexural strength, before use for the construction of semi-flexible pavement surfacings. Among these properties, one significant consideration is the flowability (or fluidity) of the cement grout, which is required to be fully penetrated through the depth of the porous asphalt surface layer without excessive compaction and vibration [93]. The second important consideration is the grout’s compressive strength, which significantly influences the final strength and durability properties of semi-flexible mixtures. 

### 6.1. Flowability of the Cement Grouts

**Figure 6:**
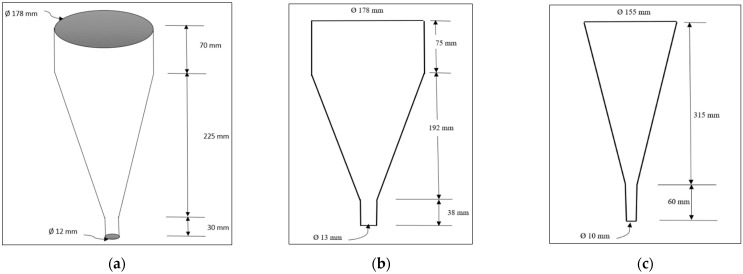
Flow cones with different geometries: (**a**) Malaysian flow cone (**b**) ASTM flow cone (**c**) Marsh flow cone (dimensions not-to-scale).

Highly flowable cement grouts are required for semi-flexible pavement applications, so that they can be easily infiltrated throughout the depth of the layer. The flowability or fluidity of cement grout is dependent on cementing materials, water–cement ratio, and superplasticizer. Due to its highly flowable nature, the flowability is measured by using a flow cone apparatus. In flow cone apparatus, the required amount of grout (soon after preparing) is poured and allowed to flow out from the apparatus. The time required to empty the flow cone is measured in seconds and recorded as flow of the cement grouts. The higher the time of flow means the flowability or fluidity of grout is lower and it will be difficult to penetrate the voids in the mixture. The standard flow time, however, depends on the geometry and size of the flow cone used. Three different sizes of cones are being used and are shown in Figure 6. The Malaysian flow cone (Figure 6a) required 1000 mL of grout and the flow time is in the range of 11 s to 16 s [56,83]. Using a Malaysian flow cone, the cement grouts with a flow of 11–16 s have sufficient fluidity and are suitable for grouting SFP surfacings. On the other hand, the ASTM flow cone (Figure 6b) required 1725 mL grout and flow time is in the range of 10 to 14 s. The Marsh flow cone (as shown in Figure 6c) also requires 1000 mL cement grout; however, due to changes in geometry, flow time is in the range of 8 to 10 s [36].

Flow of cement grout has been evaluated by changing the w/c ratio, supplementary cementing material (SCM), superplasticizer, and other additives. Superplasticizer can enhance the flowability at a relatively low w/c ratio, while achieving the desired strength properties [46,94]. A study reported improvement in compressive strength and flowability with the addition of 1% superplasticizer, whereas, increasing the water-to-cement ratio causes a reduction in the strength of grouts [95]. Afonso et al. (2016) reported low strength at a high w/c ratio and the incorporation of styrene–butadiene admixtures produces weak grouts [46]. However, stronger grout at a relatively low w/c ratio with enhanced compressive strength was achieved by adding milled glass and polycarboxylate superplasticizer [46]. 

Zhang et al. (2016) replaced cement with fly ash and mineral powder (0 to 20%), while varying the w/c ratio from 0.48 to 0.63 to formulate cement paste for semi-flexible pavement applications. The conclusion from the study indicate that increasing the water-to-cement ratio caused a significant increase in the flowability. Similarly, the increase in fly ash content causes an increase in flowability. However, the addition of mineral powder initially causes a reduction in fluidity, and then an increase was witnessed. The optimum dose of fly ash and mineral powder was recommended as 10% and at water-to-cement ratio of 0.56–0.58 for grout suitable for semi-flexible pavement applications [49]. Flow cone and flow table tests are the most common techniques used to determine the fluidity of cementitious grouts. The effect of Polycarboxylic-ether polymer superplasticizers and silica fume on fluidity and strength properties of the grout was studied, and it was concluded that sufficient workability of the grout was achieved by a 0.30 water-cement ratio with 2.0% superplasticizer and 5% silica fume by weight of OPC [96].

Therefore, based on the observations from the above-cited literature, it is important to evaluate the flowability (or flow) of cementitious grouts when designing the cementitious grouts for semi-flexible pavement surface applications. The desired flowability of grouts (while not compromising on compressive strength) plays an important role in the full penetration of voids throughout the depth of the open-graded asphalt layer. 

### 6.2. Strength Properties of Cement Grouts

Another important property of cement grout that needs to be considered for SFP surfacing is compressive strength. It mainly depends on adding SCM and water-to-cement ratio and other additives/admixtures. It was found in the literature that different researchers have used mineral powders, gypsum, silica fume, fly ash, and ground granulated blast furnace slag (GGBS) for producing cement grouts for semi-flexible pavement applications. Zhang et al. (2016) reported a significant reduction in compressive strength while increasing the water-to-cement ratio from 0.48 to 0.63. However, flexural strength was slightly reduced [49]. Therefore, cement grout for semi-flexible pavement applications is required to be introduced with superplasticizer and SCM (such as fly ash, silica fume etc.) to enhance the strength and flowability at a relatively low water-to-cement ratio. 

The combination of silica fume in addition to superplasticizer can produce grouts with sufficient fluidity and strength properties that can be recommended for semi-flexible pavement surfacings. Hence, silica fume with 5% replacement and 2.0% polycarboxylate-based superplasticizer at a 0.30 w/c ratio gives fluidity of 15 s (as determined by the Malaysian flow cone) and compressive strength at 28 days of 92.5 MPa [96]. Therefore, this grout can be recommended to produce high-strength cement grouts for semi-flexible pavement surfacings that can be used in pavements exposed to a heavy vehicular load. Moreover, the compressive strength of 57.5 MPa and 95.5 MPa at 7 and 28 days, respectively, was obtained from this grout. Furthermore, with the same grout composition, the highest flexure strength, 6.7 MPa at 7 days and 9.1 MPa at 28 days, was achieved [96]. Some commercial cement grouts are also available for the construction of semi-flexible pavement surfacing applications in the UK and Canada, etc. However, the compositions of those cement grouts are unknown. The details of some commercially available cement grouts are presented in Table 8. Furthermore, various other cement grouts that have been used in the literature for semi-flexible pavement surfacing are also presented in Table 9. 

## 7. Performance Evaluation of Semi-Flexible Mixtures

Several physical and performance properties have been used to investigate the suitability of semi-flexible mixtures and their comparison with the conventional flexible pavement. It includes the grouting ability, indirect tensile strength, moisture resistance, thermal expansion coefficient, flexural strength, fuel spillage resistance, stiffness modulus, fatigue life, and rutting properties of semi-flexible pavement surfacing materials [40,42,46,58,70,78,85,105]. Some of these properties that have been previously investigated and their corresponding testing methods are summarized in the following sub-sections.

### 7.1. Grouting Ability

The grouting ability of the semi-flexible mixture is measured by determining the degree of grout saturation. It indicates how many air voids (pores) of the open-graded asphalt mixture are being filled with the grouts [62]. It is one of the important parameters that may influence a semi-flexible mixture’s performance when subjected to heavy traffic loading and poor weather conditions [63,64]. According to the Chinese standards, the degree of grout saturation should be ≥92% [64]. The degree of grout saturation is not only a function of air voids in the porous asphalt mixture, but is also dependent on the voids’ morphological characteristics, size, and structure, which directly affect the interconnectivity of voids in the porous asphalt mixture [65]. Unfortunately, there is still no standardized guideline available that indicates a specific range of degree of grout saturation. However, some specific limits are defined in the literature. Studies conducted by Luo et al., 2018 and Zhong et al., 2020 suggest that the degree of grout saturation is ≥96% [62,100]. However, Shaoke et al. (2018) mention it as ≥92% in their article [64]. Similarly, some researchers suggest that the degree of grout saturation is in the range of 94 to 96% [54,63,105]. Therefore, based on the above-cited references, it could be suggested that the degree of grout saturation is in the range of 94–96%, which would justify the interconnectivity of voids and uniform grout penetration through the surface layer. 

### 7.2. Indirect Tensile Strength and Moisture Resistance

The indirect tensile strength (ITS) test has been used to determine the tensile strength of semi-flexible mixtures, as well as moisture resistance when conditioned in water. The ITS of semi-flexible surfacing/grouted macadam also depends on the type of aggregate gradations used in the mix design. In a study, the ITS of grouted macadam was determined for six different aggregate gradations (AG) based on different nominal maximum aggregate sizes (NMAS) [106], and the results are shown in Table 10. 

Similarly, three different open-graded asphalt gradations were adopted from ASTM, New Jersey, and New Zealand specifications, and indirect tensile strength was determined. The ASTM gradations depict the highest ITS value of about 1.6 MPa at 4.5 bitumen content, followed by New Jersey with an ITS value of about 1.4 MPa. The lowest ITS value was obtained for New Zealand, with about 1.35 MPa [107]. However, no data are available related to air voids obtained for these selected gradations. 

Furthermore, the ITS test can also be used to evaluate the moisture susceptibility of semi-flexible mixtures. The ITS is also determined for the samples conditioned in a water bath at 60 °C for 24 h before testing. The samples are then brought to a test temperature of 25 °C. The ITS obtained at these conditions can be represented as ITS_wet_. The ratio of ITS_wet_ and ITS_dry_ expressed as tensile strength ratio (TSR) represents the material resistance to moisture damage. In a study, a TSR of 0.97 was obtained for grouted macadam, whereas TSR for HMA mixtures was 0.89. The TSR results show that grouted macadam has superior performance against moisture damage than HMA [97]. Another study also concluded that semi-flexible mixtures using a cement–asphalt emulsion paste as a grout have higher ITS values and showed better resistance to moisture damage in terms of higher TSR values [102]. These studies indicated that grout phase materials in semi-flexible mixtures have an encouraging effect and are the appropriate coating in the asphalt mixture mastic [63]. The semi-flexible mixtures (grouted macadam) gain the ITS of 2.33 MPa, whereas bituminous concrete with only 0.99 MPa [108]. Furthermore, semi-flexible mixtures exhibit enhanced resistance to moisture and freeze-thaw damage compared with bituminous concrete in terms of TSR values [62,108]. The indirect tensile strength of semi-flexible mixtures also increases with an increase in curing age, due to the involvement of the cement grout’s hydration process. 

### 7.3. Compressive and Flexural Strength

It is usually required to determine the compressive and flexural strength of semi-flexible pavement surfacing after a certain curing period; however, it is not required in conventional flexible pavement material design. The semi-flexible pavement surfacing material behaves between flexible and rigid pavement materials, depending on the testing method and test conditions (most notably, temperature). Therefore, due to the inclusion of cement grout, semi-flexible materials have a flexural behavior under the application of traffic load. 

A study was conducted using waste tire rubber powder (3, 4, 5%) as an additive and activated natural zeolite (0 to 25%) as a cement replacement in cementitious grouts for semi-flexible pavement surfacing [2,109]. The increasing percentage of zeolite mineral from 0 to 15% replacement contributes to the significant increase in compressive strength, while a slight decrease was witnessed beyond 15%. The reaction of calcium hydroxide (produced due to the hydration of cement) with silica (present in zeolite mineral) results in calcium silicate hydrate formation. This contributes to an increase in the cement grouts’ density and, hence, causes an increase in the compressive strength of semi-flexible mixtures. The increase in waste tire rubber powder also causes improvement in compressive strength [109]. The behavior of improvement in flexural strength due to waste tire rubber and zeolite mineral was observed as quite similar to the compressive strength. The flexural strength of semi-flexible mixtures increased with increasing zeolite content up to 15% [109]. 

Gradation type, bitumen content, and curing age also significantly influence the compressive and flexural strength of semi-flexible mixtures. Sunil, 2020 [107] uses ASTM, New Jersey, and New Zealand-based porous asphalt gradation to produce semi-flexible mixtures. Bitumen content of 4%, 4.5%, and 5% by weight of aggregates was used to prepare porous asphalt mixtures for all three gradations. Optimum compositions of cement grouts with a cement:sand ratio of 1:1, w/c ratio of 0.45, and superplasticizer dose of 0.4% were used for all mixture combinations. It was reported that maximum compressive and flexural strength was at 4.5% bitumen content. Similarly, the gradations adopted from ASTM showed higher compressive and flexural strength at all bitumen contents and curing ages, followed by the New Jersey gradation. Moreover, all the mixtures’ compressive and flexural strengths significantly increased with increasing curing age, from 1 day to 28 days [107]. 

### 7.4. Stiffness

The stiffness of the asphalt material and grouted macadam is considered one of the important input parameters for the structural design of pavement layers. The stiffness property indicates how the traffic load is distributed effectively to underneath layers. The stiffness property is used in ranking different asphalt mixtures. The stiffness modulus is also used to evaluate the structural behavior of pavement [110]. 

Stiffness properties of semi-flexible materials or grouted macadam depend on the type of binder, mix design of the asphalt mixture, type of grouting materials, and testing conditions (i.e., temperature, loading cycle, frequency). It is a well-known fact that HMA behaves as a viscoelastic material, and the properties are changed with a temperature change. However, semi-flexible behaves differently depending on test type and conditions. It is evident from the literature that the stiffness of semi-flexible pavement surfacing materials also changes with temperature changes. The stiffness decreases with an increase in test temperature, and vice versa [36,40]. Therefore, semi-flexible pavement surfacing materials also behave visco-elastically in nature, from the perspective of the stiffness modulus. 

### 7.5. Fatigue Life

The fatigue life of semi-flexible pavement surfacing depends on many factors, such as gradation type, properties of the binder used, the combination of cement grouts and types, and input conditions of fatigue testing. In a study, ASTM, New Jersey, and New Zealand-based porous asphalt gradation were used to produce semi-flexible mixtures. Bitumen contents of 4%, 4.5%, and 5% by weight of aggregates were used to prepare porous asphalt mixtures for all three gradations. Optimum compositions of cement grouts with a cement:sand ratio of 1:1, w/c ratio of 0.45, and superplasticizer dose of 0.4% were used for all mixture combinations. The semi-flexible mixtures with ASTM gradations showed the highest fatigue life at different stress ratios (10, 20, and 30%) compared with New Jersey and New Zealand gradations [107]. 

**Figure 7:**
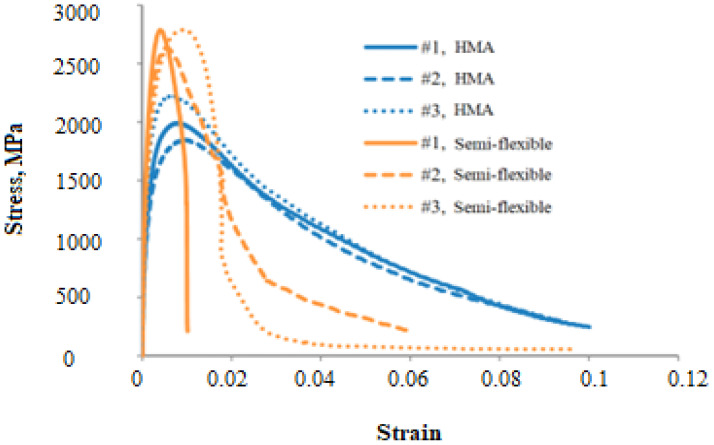
Stress–strain relationship of fatigue test results [97].

However, the application of semi-flexible pavement surfacings (grouted macadam) is quite different from conventional HMA. In order to compare the fatigue life of semi-flexible mixtures with conventional HMA based on strain, a study has shown that semi-flexible pavements have a lower fatigue life compared with HMA [97]. This is due to the brittle behavior of the semi-flexible material because of the cement grouts, which cause fatigue failure at low strain compared with conventional flexible pavement materials. Semi-flexible mixtures can withstand higher maximum stress compared to HMA. However, a sudden and abrupt fall in stress happened in semi-flexible mixtures, as shown in Figure 7 [97]. However, a study conducted by Oliveira et al. (2008) based on the strain-controlled test method, concluded that semi-flexible pavement surfacing has a higher fatigue life compared with HMA [111]. 

The resistance to fatigue failure of semi-flexible mixtures may be lower than conventional asphalt mixtures due to higher stiffness and brittleness. However, the overall fatigue performance of semi-flexible pavement surfacing materials should be investigated based on their application. It also depends on what the surface application will be used for. What are the loading scenario and design life? The tensile strain response of semi-flexible mixtures in the layer can significantly be reduced due to a high modulus and can compensate for its fatigue performance [97]. Consequently, a comprehensive pavement layer of semi-flexible mixtures is structurally evaluated considering field conditions of traffic loading, material characteristics, and environmental conditions. 

Several types of laboratory tests are available to investigate the fatigue resistance of asphalt mixtures. These include a two-point bending test on trapezoidal specimens or prismatic-shaped specimens, a three-point bending test on prismatic-shaped specimens, a four-point bending test on prismatic-shaped specimens, and an indirect tensile test on cylindrical-shaped specimens. Moreover, based on the loading mode, fatigue tests are divided into two categories: control stress mode and control strain mode. In control stress mode, constant stress is applied, and fatigue life is defined as a complete failure of the specimen. However, in control strain mode, the complete failure of specimens is unlikely to occur. Therefore, fatigue life is defined as when the stiffness dropped to half of the initial value [112,113]. 

### 7.6. Rutting

Semi-flexible pavement surfacing materials showed superior performance against rutting, and the deformation could simply be negligible. Few studies evaluated the rutting (permanent deformation) in semi-flexible pavement surfacing material and the results were very satisfactory. Due to a higher stiffness modulus, semi-flexible pavements experienced no permanent deformation throughout their design life. 

A study concluded that a semi-flexible mixture with New Zealand and ASTM gradation at 4.5% bitumen content showed quite similar deformation results and completed 20,000 cycles without failure and negligible deformation was witnessed [107]. Similarly, semi-flexible mixtures produced from different grouts exhibit the smallest rut depth and the highest dynamic stability of more than 13,000 times/mm at 60 °C temperature [101]. Semi-flexible mixtures depict high-temperature stability compared with HMA mixtures [101].

The dynamic stability and, hence, the rutting resistance of grouted macadam was recorded as 2.5-times higher than dense-graded asphalt mixtures. The dynamic stability of grouted macadam experienced more than 11,000 passes/mm, while asphalt concrete less than 5000 passes/mm [100]. Different laboratory tests are available to determine the extent of rutting in asphalt mixtures. These include the Asphalt Pavement Analyzer (APA), Hamburg Wheel-track Test, Asphalt Mixture Performance Tester (AMPT), and High-Temperature Indirect Tensile Test [114]. APA is used to measure rutting directly by producing deformation under an applied load. In this test, the traffic load is simulated by rolling a loaded wheel at a high temperature over an inflated (specified pressure) rubber hose [114,115]. The Hamburg Wheel-track Test is also used to measure rutting directly under the application of wheel loads. In this test, the traffic load is simulated by moving a loaded steel wheel directly on the mixture’s surface. The Hamburg Wheel-track test can also be used to assess the moisture sensitivity of asphalt mixtures [114,116]. 

## 8. Compatibility of Cement Grout with Asphalt Mixture (Microstructure Characterization)

In conventional asphalt mixtures, the aggregate acts as a skeleton that transfers the load, while the asphalt acts as a binding/glue medium that binds aggregates together. However, in semi-flexible mixtures, the situation is a little bit complex, because it is a composite material with at least three phases (aggregate, bitumen matrix, and cement grout). Cai et al. (2019) evaluated the reinforcement mechanism of a semi-flexible pavement mixture and come to the conclusion that some parts of the cement grouts have an additional interlocking effect with the aggregate skeleton [117]. 

Furthermore, the interlocking effect of the skeleton could be improved by increasing the porosity of open-graded asphalt mixtures and, as a result, produces a positive effect on the reinforcement of semi-flexible mixtures [98,117]. The interlocking effect of grouting cement to produce an additional skeleton is much related to the interconnected voids within the open-graded asphalt mixtures [117]. A study was conducted to observe the interface of asphalt and cement grout in semi-flexible mixtures, which concluded that increasing porosity and gradation type improves the contact of grout and asphalt concrete mixtures [99]. Zhong et al. (2020) utilized latex-modified cement mortar for grouting open-graded asphalt concrete to produce semi-flexible pavement surfacing. A pull-out test was used to investigate the grout and asphalt concrete interface. The results indicate that latex-modified mortars proved to have good adhesion with asphalt concrete when compared to adhesion between the control cement grout and asphalt concrete [100].

**Figure 8:**
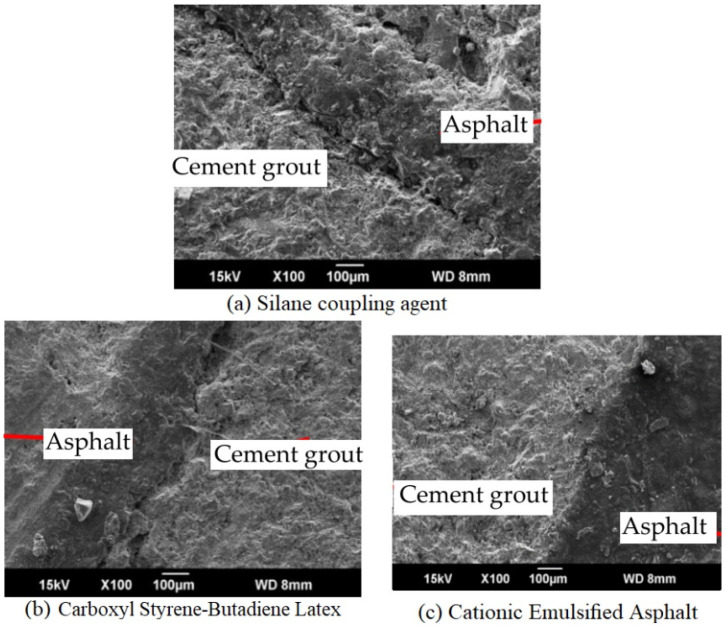
Effect of interface modifiers on grout–asphalt adhesion [101].

Using interface modifiers in cement grouts can also result in improved adhesion or compatibility of cement grout with asphalt concrete. Xu et al. (2020) [101] investigated three interface modifiers (namely, silane coupling agent, carboxylated styrene–butadiene latex, and cationic emulsified asphalt) in cement mortar and investigated the interface bonding along with other performance properties, as shown in Figure 8. They concluded that the cationic emulsified asphalt interface modifier (Figure 8c) produces a good adhesion bond with asphalt and no obvious cracks were found during the microscopic study. While the other interface modifiers showed some cracks at the joint of grout and asphalt, as shown in Figure 8a,b [101].

**Figure 9:**
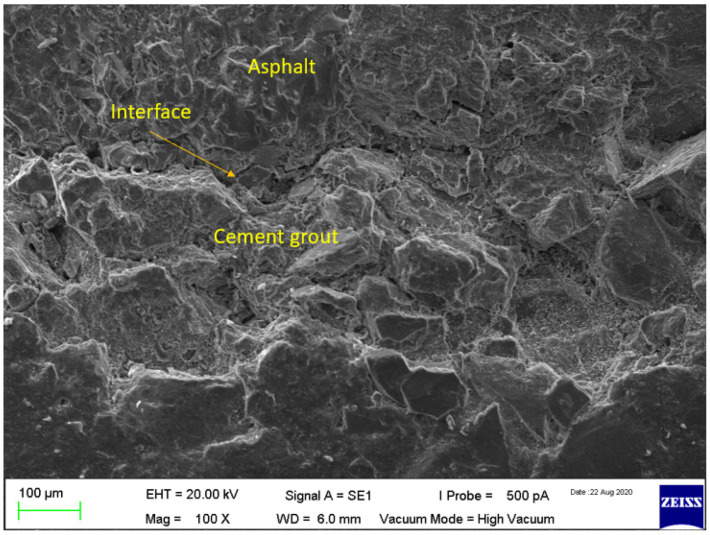
SEM micrograph of semi-flexible with control cement grout.

However, despite using modified grouts or interface modifiers, the interface of cement grout and asphalt concrete is still the weak spot and may lead to cracking after the application of an external load or environmental stresses. The semi-flexible pavement surfacing (grouted macadam) is a composite structure, having at least three phases (such as aggregates, bitumen, and cement grout). The cement grout is injected in the open-graded asphalt mixture (when it is cooled) and, therefore, there is a possibility of compatibility (adhesion) issues between the cement grout and asphalt mixture. To investigate the compatibility and adhesion between cementitious grout and asphalt mixture, SEM analysis was performed. Two types of the mixture were investigated: semi-flexible with a control grout (SFP-CN) and semi-flexible with an irradiated PET grout (SFP-IrP). The micrographs from the SEM test of the control grout-based semi-flexible specimen is shown in Figure 9.

**Figure 10:**
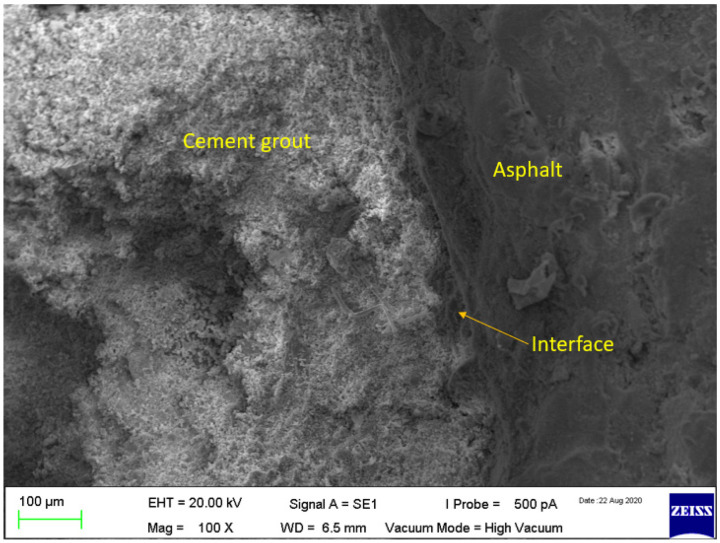
SEM micrograph of semi-flexible with Irradiated PET-based grout.

It can be observed in Figure 9 that there are some micro-cracks at the interface of the asphalt mixture. However, no obvious cracks are found in semi-flexible specimens grouted with irradiated PET (Figure 10). It indicates that the adhesion bonding between the cement grout and asphalt mixture has been improved. The cracks in SFP-CN may be attributed due to the shrinkage of cementitious grout. Moreover, the uneven morphology and the microcracks may be induced during the cutting and polishing of specimen preparation for SEM analysis [118]. The improved adhesion between the cement grout and asphalt mixture in SFP-IrP is attributed to its better compatibility compared to SFP-CN.

## 9. Conclusions

According to the reviewed articles regarding semi-flexible pavement surfaces, it can be summarized that this type of pavement layer can produce a high-performance pavement surface. Following are the main conclusions drawn from this review:

- It can be concluded that the durability and performance properties of semi-flexible pavement surfaces largely depend on the mix design of the porous asphalt skeleton and composition of cementitious grouts. The selection of aggregate gradation and type of bitumen in porous asphalt mixtures and their effect on final semi-flexible specimens have been presented. Similarly, the effect of different compositions of cementitious grout and other additives/admixtures on the performance of semi-flexible mixtures have been explained.
- Single-size aggregate is used to achieve the required porosity of porous asphalt mixtures. A single aggregate size in the range of 8–12 mm nominal size can be used in porous mixtures to achieve the void ratio of 25–35%. Furthermore, polymer-modified binder can be used in the porous asphalt skeleton to improve its resistance against high and low-temperature performance. Municipal and/or industrial wastes/byproducts could be a better choice in binder modification towards performance improvement and environmental sustainability.
- The grouts are required to be highly flowable to penetrate through the porous asphalt skeleton. The flow value of 11 to 16 s is recommended while using the flow cone of the size used in ASTM C939. Depending on the composition of grouts, a w/c ratio of 0.45–0.65 could be used to achieve the required fluidity of cementitious grouts. Moreover, superplasticizer (0 to 2%) is used to achieve high fluidity at a relatively low w/c ratio (0.30 to 0.40). Other pozzolanic and cementitious products (such as fly ash, silica fume, fine limestone, and ground granulated blast furnace slag, etc.) could be used to achieve medium-to-high-strength grouts and, hence, medium-to-high-strength semi-flexible mixtures, and achieve the sustainability goals. Fly ash, silica fume and ground granulated blast furnace slag up to 10%, 5%, and 10%, respectively, can be used to replace cement, while achieving the required strength properties.
- The performance of cementitious grout is significantly related to the performance of semi-flexible mixtures. Therefore, the grouts are carefully designed to produce high-performance semi-flexible pavement surfaces.
- The fatigue life of semi-flexible mixtures can be evaluated using two-point or four-point bending fatigue tests. However, a four-point bending fatigue test could give better and more reliable results compared to the two-point fatigue test, because the failure of beams occurs in the uniform bending moment area.
- It is recommended to use interface modifiers to enhance the bonding between the asphalt mixture and cement grouts that help in reducing cracks in the pavement.
- Further study could be conducted on utilizing industrial/municipal wastes and other byproducts as a cement replacement in cementitious grouts for semi-flexible layers. It is a known fact that the production of cement releases carbon dioxide both directly and indirectly. Hence, there is a need for the development of durable, sustainable, and cost-effective cementitious grouts for semi-flexible pavement surfaces by utilizing a large percentage of municipal and industrial wastes/byproducts.

## Figures and Tables

**Table 1:** Development and purpose of grouted macadam in the past.

Reference	Country	Brand Name	Purpose of Construction
[33,35]	France	Salviacim	To provide resistance against waste oils, fuels, and abrasion
[36]	United States	Resin-Modified Pavement (RMP)	Airport taxiways, aprons, parking lots
[37,38,39]	Europe	Hardicrete Heavy Duty Surfacing. Worthycim Heavy Duty Paving. Confalt	Heavy-duty surface construction
[40]	Japan	RP-Pavement (Rut Proof Pavement)	Heavy-duty surface construction
[35]	France	combi-layer	Heavy-duty surface construction

**Table 2:** Effect of gradation type on performance properties of semi-flexible pavement [58,59].

Gradation Type	Modulus of Elasticity (MPa)	Compressive Strength (MPa)
G1	18,000	6.09
G2	17,600	5.10
G3	17,200	4.70

**Table 3:** Gradation adopted by Saboo et al. (2019) [51].

Sieve Size (mm)	Passing Percentage						
	Boundy (1979) Mix 1	BSI (1987) Mix 2	Anderton (2000) Mix 3	Densiphalt Type 8 Mix 4	Densiphalt Type 12 Mix 5	Setyawan (2003) Mix 6	Hou (2017) Mix 7
19	100	100	100	100	100	100	100
12.5	95	100	62.8	100	100	100	
9.5	35	92.5	51.5	95	95	90	44
6.3	10	17.5	35	30	12	38	
4.75	6	5	17.6	20	10	8	10
2.36	3	1	11.1	10	8	5	
1.18			7.3	5	5	3	
0.6			6.6				2
0.3			2.4				
0.15			1.2				
0.075			1.1				
Air Voids	31–32%	33–34%	27–31%	33–36%	32–35%	29–32%	30–32%
Binder Content range, % (Optimum, %)	2–3.5% (4%)	2–4% (3.5%)	2–4.5% (3.5%)	2–4.5% (4%)	2–4.5% (4%)	2–4% (4%)	2–3.5% (3.5%)
ITS (kPa)	110.46	113.35	116.52	118.83	114.42	123.32	109.85

**Table 4:** Acceptance/rejection criteria for the mixtures.

S. No	Properties of Mix	Acceptable Criteria	Initial Selection of Mixes	Final Selection
1	Draindown test	<0.3%	Mix 1, 3.5% Mix 1, 4.0% Mix 2, 3.5% Mix 3, 4.0% Mix 3, 4.5% Mix 4, 4.0% Mix 4, 4.5% Mix 5, 4.0%	Densiphalt type 12 (Mix 5) with 4.0% bitumen Densiphalt type 12 (Mix 5) with 4.5% bitumen BSI (Mix 2) with 4.0% bitumen
2	Air Voids	25% to 35%		
3	Voids in coarse aggregate (VCA) ratio	<1%		
4	Permeability	>100 m/day		
5	Cantabro loss	<50%		
6	Indirect Tensile Stiffness (ITS)	N/A		

**Table 6:** The recommended range of additives and w/c ratio for grout composition [70].

Cement Paste Compositions and Additives	Range of Variables Used	Recommended Values	Evaluated Properties of Grouts
Water–cement ratio	0.51–0.57	0.55–0.57	Fluidity Ductility Drying shrinkage Compressive and Flexural strength
TH-928 polycarboxylate superplasticizer	0–1.0 (%)	0.5–1.0 (%)	
UEA expansion admixture	0–12 (%)	10%	
ZY-99 Triterpenoid saponins air-entraining agent	0–0.012 (%)	0.008%	

**Table 7:** Recommended value range for High-Performance Cement Pastes (HPCP) [70].

HPCP Composition/Type	H1	H2	H3
w/c ratio	0.56	0.56	0.56
TH-928 polycarboxylate superplasticizer	0.5%	0.5%	1.0%
UEA expansion admixture	10%	10%	10%
ZY-99 Triterpenoid saponins air-entraining agent	0.008%	0.0%	0.008%

**Table 8:** Technical data of commercially available cement grouts.

Type of Cement Grout	Manufacturer Name/Country	Compositions	Properties	Application	Remarks
Densiphalt mortar	Densit^®^/UK	Composition Not Known Water = 16.2% by weight of powder	Compressive Strength 1-day = 50 MPa 7-day = 80 MPa 28-day = 110 MPa Density = 2200–2250 Kg/m^3^ Setting time = 7–9 h	Warehouses, distribution centers, goods terminals, production floors, Harbors, container terminals, warehouse facilities, Bus stops and terminals, roads for heavy traffic, road crossings, Aircraft aprons, taxiways, refueling pads, taxi-holding positions	These are the few commercially available cement grouts for semi-flexible pavement applications. Most of these grouts did not provide the compositions of cement grouts. Few grouts are provided with technical requirement. Among these grouts, nobody used waste PET and FA in the formulation of cement grouts.
DuraTough	Lafarge/Canada	Composition Not Known	Standard grout: Compressive Strength 7-day = 35 MPa 28-day = 60 MPa High-Strength grout: Compressive Strength 7-day = 60 MPa 28-day = 100 MPa Flexural Strength 28-day = 2.5–3.0 MPA	Bus stops and transit stations, turning lanes and intersections, high-volume and heavy-load roadways, tunnels and hard-to-access areas Apron areas, taxiways, fueling/deicing areas, hangar bays, loading bays	
Hardicrete	Miles Macadam Ltd./UK	Composition not known	No Specifications	Bus stations, waste sites and transfer stations, ports, airfields, and industrial facilities	
ULTICRETE	TARMAC A CRH company/UK	Composition not known	No Specifications	aircraft refueling areas, container ports, and distribution centers.	
CONFALT^®^ mortar	Contec ApS/ Denmark	Portland Cement = 45–75% Silica sand = 25–45% Iron Oxide = 0–5% Amorphous Silica = 5–25%		Storage Facilities, Industrial Flooring, Cross Sections, Container Terminals, Airports, Multistorey Parking	

**Table 9:** Description of cement grouts from the literature.

Composition of Grouts	Strength Properties of Cement Grouts	Concluding Remarks	References
0.50 w/c ratio, 23% fly ash and 2% superplasticizer	Not determined	Semi-flexible mixtures compared to HMA Rut resistance Resistance to moisture damage Lower fatigue life Better thermal resistance	[97]
0.31 w/c ratio, pre-designed grout (JGM-301)	Compressive Strength: 34.5 MPa at 3 d curing and 42.3 MPa at 28 d curing	It was recommended that semi-flexible pavement surfacings with this grout could be used for heavily loaded pavements.	[98,99]
0.72 w/c ratio, 20% sand, 10% filler and 0–2.4% Latex	Compressive Strength: 21.6 MPa to 24.1 MPa compressive strength and 1.65 MPa to 3.54 MPa flexural strength at 7d curing	Latex in cement mortar has a positive effect on compressive and flexural strength, while negatively impacting fluidity. Semi-flexible mixtures compared to HMA Rut resistance Resistance to moisture damage Better fatigue life Better thermal resistance Poor resistance to brittle cracking	[100]
Three interface optimizers were used: 0.25 to 0.75% Silane coupling agent, 5 to 15% Carboxyl styrene-butadiene latex, and 5 to 15% Cationic emulsified asphalt	7-days compressive Strength: (1) 42.98 MPa for OPC (2) 34.65 MPa, 36.85 MPa, 33.61 MPa for Silane coupling agent (3) 25.70 MPa, 29.70 MPa, 26.15 MPa for Carboxyl styrene-butadiene latex (4) 37.79 MPa, 33.82 MPa, 31.53 MPa for Cationic emulsified asphalt	With interface optimizers Improvement in resistance to Drying shrinkage Improvement in interface bonding Improvement in resistance to low temperature cracking improve high temperature stability and moisture resistance.	[101]
0.40 w/c ratio, 10% silica fume, 2% Superplasticizer, 0.04% aluminite powder (AP), 0.2% viscosity modifying agent (VMA) ≥ VMA/C, 20–60% Asphalt Emulsion (AE)/cement binder AE/C	7-days compressive Strength: CP = 26 MPa CAEP20% = 13 MPa CAEP40% = 11.3 MPa CAEP60% = 10.5 MPa 28-days compressive Strength: CP = 28.5 MPa CAEP20% = 19 MPa CAEP40% = 17.8 MPa CAEP60% = 17 MPa	Asphalt emulsion reduces compressive strength, whereas it improves interface bonding Rutting resistance is improved Better moisture resistance Poor resistance to low temperature	[102]
2–4% Naphthalene-based Superplasticizer and 0.4 to 0.6 w/c ratio and 23% Sand (different size) by weight of cement	7-days compressive Strength: For sand size < 0.6 mm gives maximum of 31.65 MPa compressive strength For sand size < 1.18 mm gives maximum compressive strength of 33.75 MPa For sand size 2.36 mm gives maximum compressive strength of 36.69 MPa	Grouts with 0.54 w/c ratio and 2% SP as optimum combination achieving the compressive strength of 24.25 MPa.	[103]
0.5 to 2% Polycarboxylate Superplasticizer, 0.4 to 0.50 water-to-cement ratio and 23% sand (with different size)	7-days compressive Strength: 13.15 MPa to 29.10 MPa (for sand size < 0.6 mm) 16.38 MPa to 30.39 MPA (for sand size < 1.18 mm) 18.45 MPa to 31.20 MPA (for sand size < 2.36 mm)	The optimal composition of grout: 0.48 w/c ratio of 0.48, and 1% SP with 23.46 MPa compressive strength of grout	[103]
0.72 water-to-cement ratio, 20% sand, 10% filler, and 1.2 to 2.4% latex powder (LP)	7-days Compressive Strength: Maximum compressive strength of 24.1 MPa at 1.2% LP and maximum flexure strength at 3.54 MPa at 2.4% LP	Fluidity decreases with increasing latex powder. 1.2% LP has highest compressive strength, whereas maximum flexural strength is achieved at 2.4% LP LP modified semi-flexible mixtures showed improved resistance to high temperature Poor resistance to fatigue and low-temperature cracks compared to HMA	[62]
0.63 w/c ratio, 10% mineral filler, 20% Sand, and 2–12% Carboxyl Latex (CL)	11.20 to 20.76 MPa of 7-days Compressive Strength and 18.56 to 31.43 MPa of 28-days Compressive Strength 4.95 to 28.74 MPa of 7-days Flexural Strength and 5.74 to 6.49 MPa of 28-days Flexural Strength	The addition of CL causes reduction in compressive strength, while improvement in flexural strength Optimum content of CL obtained was 8%. Rutting, low-temperature cracking, moisture damage and fatigue resistance of modified CL was improved compared with controlled grout	[72]
0.60 water-to-cement and cement/sand ratio of 1/0.5	10.40 MPa of 3d compressive strength and 30.7 MPa of 28d compressive strength 3.3 MPa and 7.3 MPa of flexural strength at 3d and 28d curing	Semi-flexible specimens showed better high-temperature performance, improved low-temperature cracking, moisture resistance compared with HMA.	[104]

**Table 10:** Six types of gradations and the corresponding ITS values [106].

Designations	Description of Gradation	ITS @ 35 °C
AG1	NMAS of 19 mm and Mid-point gradation of surface dressing	1.41 MPa
AG2	NMAS of 13.2 mm and Mid-point gradation of surface dressing	1.71 MPa
AG3	NMAS of 13.2 mm and Mid-point gradation of OGPC	1.63 MPa
AG4	NMAS of 13.2 mm and Modified Mid-point gradation of surface dressing	1.57 MPa
AG5	NMAS of 9.5 mm and Mid-point gradation used by Oliveira	1.29 MPa
AG6	NMAS of 19 mm and Mid-point gradation of Bituminous Macadam	0.81 MPa

## Data Availability

Not applicable.
